# Litigation as an Underutilised Quality Feedback Indicator: A Review of Court Cases in a Small Resource-Limited Country

**DOI:** 10.7759/cureus.112231

**Published:** 2026-07-07

**Authors:** Mandreker Bahall, Krishni Bahall

**Affiliations:** 1 Cardiology, Caribbean Centre for Health Systems Research and Development, University of West Indies, St. Augustine Campus, Marabella, TTO; 2 General Medicine, San Fernando General Hospital, San Fernando, TTO

**Keywords:** healthcare, litigation, medical negligence, quality gaps, quality healthcare

## Abstract

Objective

Litigation cases provide important feedback for healthcare providers. However, this method has been underutilised. This study provides a descriptive analysis of completed court cases, exploring case characteristics, reasons for litigation, and judicial outcome. It also looks at expected standards of care based on best practice and provides feedback for quality improvement.

Methods

This is a retrospective study of court judgements on health issues from 1990 to 2023. The cases were generated from the court library services and judiciary of the Republic of Trinidad and Tobago. Data were collected using an 18-item, pilot-tested questionnaire tested on 20 cases to ensure clarity and adequate descriptive information on relevant topics for the objectives of the study. The inclusion criteria were persons of all ages and residents or citizens of Trinidad and Tobago. The exclusion criteria were cases regarding health-related but non-clinical issues, incomplete/ambiguous court reports, healthcare staff/employment matters, and cases involving motions to strike out evidence, as the full cases are not available for analysis. This questionnaire was then itemised based on patient demographics, case characteristics, outcomes and court recommendations and analysed using SPSS Statistics version 21 (IBM Corp., Armonk, NY, USA).

Results

Following the application of the inclusion and exclusion criteria, a total of 52 cases were selected to be analysed. Most litigants were female Afro-Trinidadians in the public sector. The most common reasons for litigation were delayed diagnosis/inadequate treatment (n = 23; 44.2%), followed by misdiagnosis (n = 9; 17.3%) and prescribing the wrong dosage or medication (n = 7; 13.5%). The main lawsuit outcome was of medical negligence (n = 27; 51.9%), with the obstetrics speciality having the highest number of cases convicted (n = 9/27; 33.3%). The most common patient/defendant outcome was physical in nature (n = 38; 73.1%), followed by monetary compensation (n = 18; 34.6%); disciplinary action against the healthcare provider occurred only in one case (n = 1; 1.9%). Regarding medical error in those cases that were convicted, inadequate care standards were most commonly related to inappropriate drug dosage and usage (n = 5/27; 18.5%), followed by poor listening and communication (n = 1/27; 3.7%), improper blood transfusion procedures (n = 1/27; 3.7%), and poor sterilisation of surgical instruments (n = 1/27; 3.7%).

Conclusion

Most medical negligence cases occurred in public institutions, particularly in obstetric departments, followed by emergency departments. Litigation mainly resulted from delayed diagnoses/inadequate treatments and breaching care standards. Ongoing litigation analysis is a useful tool for quality feedback and improvement.

## Introduction

Litigation is a civil procedure that provides an avenue for individuals to contest a dispute or conflict, including negligence claims, and seek redress [1]. Litigation in healthcare is expressed in different ways in different countries. Hence, it is difficult to source comparable national-level data. However, incidence varies due to factors such as poverty, underdeveloped health systems, weak infrastructure, differences in legal frameworks and regulations and civil society deficits. Generally, a rise in litigation has been noted in developing countries, such as the 400% surge in recent years in India [2]. In developed countries, however, the incidence varies. Japan noted a decline in closed medical malpractice claims since 2006, with 13,340 closed claims between 2006 and 2021, whilst the United States of America estimated 17,000 or more claims per year [3]. According to the American Medical Association, in a survey of 5,825 patient care physicians, 42.2% of the physicians had a claim filed against them at some point in their careers [4]. The total national cost of medical errors that caused injuries was estimated to be between $17 and $29 billion per year [5]. These costs included disability, loss of income and healthcare expenses. Van den Bos et al. [6] estimated this annual cost to be $17.1 billion in 2008. Litigation for medical negligence results from medical errors, such as diagnosis and treatment errors, improper staff supervision, the failure to communicate with patients or recognise complications, referral failure/delay, operating on the wrong patient or body part and surgical foreign body [7]. Hence, malpractice claims occur when a claimant, commonly a patient, 'brings a legal cause of action against his or her healthcare provider for providing (or not providing) medical treatment that falls short of the accepted medical standard of care' [8].

Litigation affects all stakeholders, including healthcare institutions, providers, patients and the public. Its impacts are broad, including financial impacts, integrity and confidence in the system. Regarding healthcare institutions, the New York University School of Law reported that medical errors increase average hospital costs, more so in the riskiest hospitals [9]. Litigation affects the patient physically, emotionally and financially. It also affects the defendant/healthcare provider emotionally and psychologically, which impacts their professional identity and integrity, giving rise to defensive medical practices. Despite the negative impact and publicity, the cases provide valuable information on demographics, nature and type of cases; case characteristics, such as reasons for lawsuits and parties involved, and case outcomes. Moreover, it provides important feedback and allows for quality assessments from the perspective of the patient and provider. Furthermore, it creates financial and political pressure to improve [10].

Whilst court cases are available in the public domain and case reports are also shared via bodies like the Medical Protection Society, litigation information is not always easily available, as some cases may not be highlighted or shared to avoid negative publicity. Additional challenges arise owing to the burden of proving negligence. This lies with the claimant, who often has difficulty procuring medical experts. Physicians are wary of testifying against colleagues in case their positions were to be reversed later. This is the 'phenomenon of the conspiracy of silence' [11]. In several developing countries, cultural practices do not reflect a litigation-oriented society, and access to justice is hampered by financial strain, long waiting periods, inadequate legal infrastructure and corruption. Few cases reach the courts. According to the National Practitioner Data Bank, from 2005 to 2009, most paid claims (96.9%) were settled outside of court, with 3.1% being judged in court [12]. Due to the nature of litigation and the potential for negativity, the few cases that end up in the courts are utilised to extract information to improve the healthcare system.

Total quality management (TQM) in healthcare exists where 'all members of an organisation participate in improving processes, products, services, and the culture in which they work’ [13]. According to Hidayah et al. [14], the use of TQM could enhance patient safety. Identifying gaps in care standards is the initial step for continuous quality improvement. The regional health authorities seek to do this through the quality department and morbidity and mortality meetings in the various clinical departments. This can also be aided through an analysis of litigation reports, which is currently lacking in the literature. Litigation exposes healthcare gaps, unfulfilled duty of care requirements, and the causal link and standards of appropriate care using the relevant bodies. Thus, it has the potential to minimise liability and litigation. Our citizens are more aware of their medical rights and are also willing to challenge the system for redress against medical negligence. This study explores court cases in a small, resource-limited country to determine the current status of medical negligence concerning patient demographics, case type, claimant type, defendant type, institution involved and court level; reasons for litigation; judicial and patient/defendant outcome; and interventions/recommendations suggested from the cases.

## Materials and methods

Study design and setting

Most medical negligence cases occurred in public institutions, particularly in obstetric departments, followed by emergency departments. Although health institutions accounted for most claims, doctors made up a significant proportion. Litigation mainly resulted from delayed diagnoses/inadequate treatments and breaching care standards. Court outcomes predominantly led to financial compensation. Expert witness information and in-depth evaluations make litigation analysis profoundly useful and should be further refined as a quality improvement feedback instrument. Greater efforts must be made by the medical fraternity to identify the type, degree, and details of negligence through litigation analyses to drive quality improvement from an individual to an institutional level.

Sample selection

**Figure 1:**
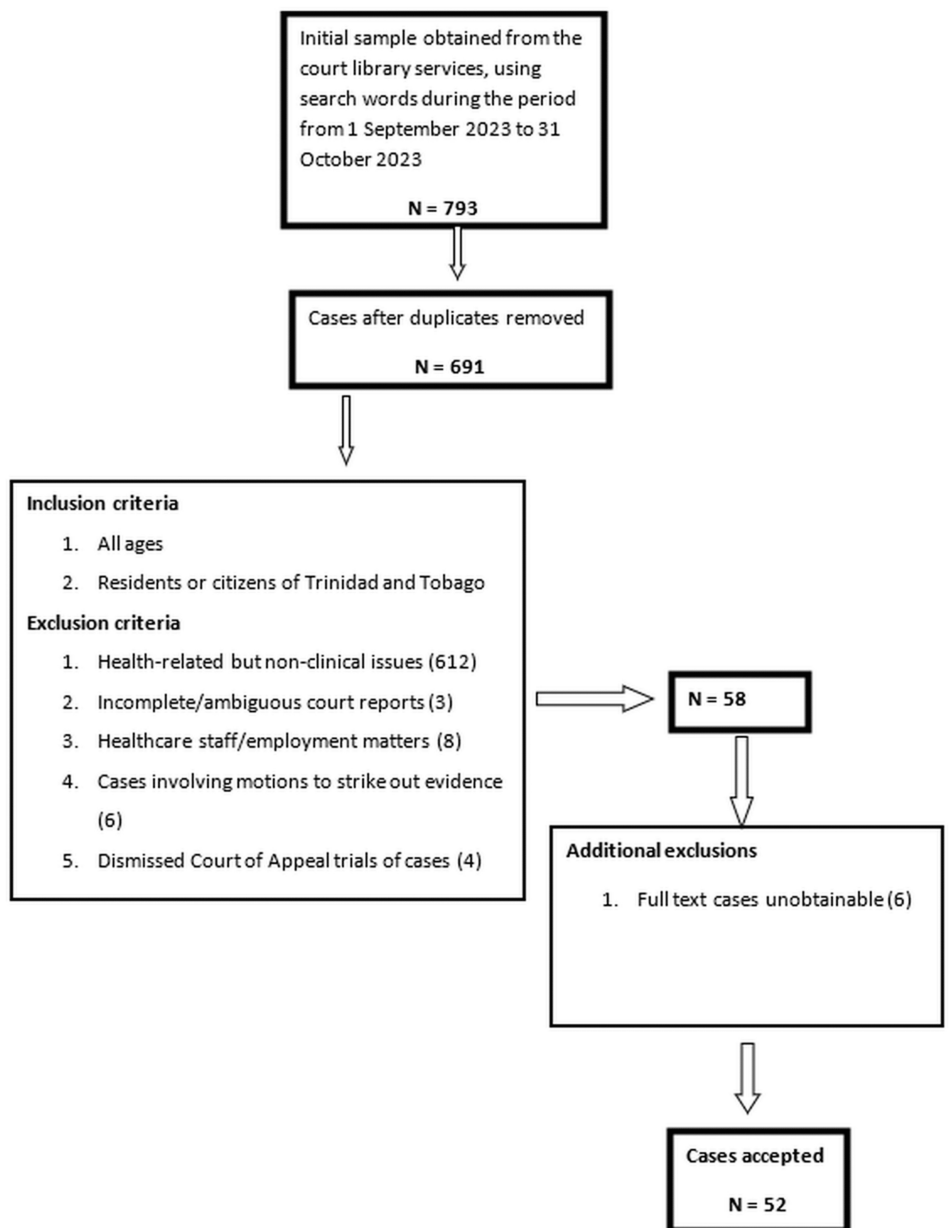
Flowchart on case selection

Judgments from law cases involving health matters from 1990 to 2023 were obtained between 1 September 2023 and 31 October 2023 from the court library services of the Judiciary of the Republic of Trinidad and Tobago, using search words such as "medical negligence", "health and medical negligence" and "health and negligence". The search strategy was similar to that used in other legal case series [15,16]. All cases were selected using an online database with the help of a legal librarian. The inclusion criteria were persons of all ages and residents or citizens of Trinidad and Tobago. The exclusion criteria were cases regarding health-related but non-clinical issues, incomplete/ambiguous court reports, and healthcare staff/employment matters, as these present no patient or clinical issue, and cases involving motions to strike out evidence, as the full cases are not available for analysis. The searches led to 793 cases, from which a final sample of 52 cases was used for data collection and analysis. This figure was obtained after removing duplicates and applying the exclusion criteria. Figure 1 provides a diagrammatic overview of the sample selection. The searches led to 793 cases, from which a final sample of 52 cases was used for data collection and analysis.

Data collection and interpretation

The data collection instrument was an 18-item questionnaire, which comprised questions on demographics (age, sex and ethnicity), socioeconomic issues (employment status), case characteristics (speciality involved, type of institution, number and nature of claimants and defendants, court involved and type and duration of case), the criminal act under investigation and case outcomes and court recommendations. The questionnaire was pilot tested on 20 cases to ensure clarity and adequate descriptive information on relevant topics for the objectives of the study. The data were collected by the authors separately and corroborated. Ambiguities in the data were clarified after discussion. The collected data were entered into Microsoft Excel version 10 (Microsoft Corp., Redmond, WA, USA) to create a database. After editing, the data were imported into SPSS Statistics version 21 (IBM Corp., Redmond, WA, USA) for data management and descriptive statistical analysis. Given the descriptive nature of this retrospective study, analysis methods included frequency and percentage distribution tables, graphs and charts, along with sample proportions or percentages for qualitative variables, and inferential statistical analyses were not undertaken.

## Results

Sociodemographics

Patients were primarily females (n = 29; 55.8%) and Afro-Trinidadians (n = 35; 67.3%). Most claimants were patients (n = 32; 61.5%), and healthcare institutions were the main defendants (n = 30 of 50 cases where relevant data were obtainable; 60%). Most cases involved public health institutions (n = 32; 61.5%) (Table 1).

**Table 1:** Characteristics of patients and lawsuits related to medical negligence (total n = 52) ^a ^Cases were not used as data were not available; ^b ^Cases were not used due to lack of clarity

Characteristics		N	%
Sex	Male	23	44.2
	Female	29	55.8
Ethnicity	Afro-Trinidadian	35	67.3
	Indo-Trinidadian	17	32.7
	Mixed	0	0
	Others	0	0
Employment Status (n = 22^a^)	Employed	13	59.1
	Unemployed	2	9.1
	Retired	0	0
	Never employed	7	31.8
	Lost job	0	0
Case Type	Civil	52	100
	Criminal	0	0
	Others	0	0
Institution involved	Public institution	32	61.5
	Private institution	20	38.5
Client type/plaintiff	Patient	32	61.5
	Relative	15	28.8
	Public Citizen	1	1.9
	Others	4	7.7
Defendant type (n = 50^b^)	Doctor	16	32
	Nurse	0	0
	Healthcare institution	30	60
	Operating system	0	0
	Support staff	0	0
	Others	4	8
Trial location	Supreme Court	0	0
	High Court	50	96.2
	District Court	0	0
	Privy Council	0	0
	Court of Appeal	2	3.8

Nature of criminal act or reason for lawsuit

The most common reason for medical negligence lawsuits was delayed diagnosis/inadequate treatment (n = 23; 44.2%). This was followed by misdiagnosis (n = 9; 17.3%) and prescribing the wrong dosage or medication (n = 7; 13.5%) (Table 2).

**Table 2:** Type of criminal act or reason for the lawsuit (total n = 52)

Characteristics	N	%
Delayed diagnosis/inadequate treatment	23	44.2
Misdiagnosis	9	17.3
Prescribing the wrong dosage or medication	7	13.5
Unexpected complications (e.g. persistent pain after surgery)	5	9.6
Leaving medical equipment/objects inside the patient	3	5.8
Lack of professional qualifications	3	5.8
Poor follow-up or aftercare	2	3.8

Percentage of cases convicted by specialty and conviction rates per specialty

The specialities predominantly involved in medical litigation cases were obstetrics (n = 15; 28.8%), others (which included anaesthetics, dentistry, radiotherapy and urology) (n = 11; 21.2%) and surgery (n = 7; 13.5%). Of these, 33.3% of conviction judgements (i.e., negligence and judgements awarded to the claimant) were from obstetrics, 25.9% from others and 14.8% from emergency medicine (Table 3).

**Table 3:** Type of specialty involved in cases and conviction data

Specialty	No. of cases	Percentage of cases	No. of cases convicted	Percentage of cases convicted	Conviction rate per specialty (%)
Primary care (family medicine)	0	0	0	0	0
Internal medicine	3	5.8	1	3.7	33.3
Surgery	7	13.5	1	3.7	14.3
Emergency medicine	6	11.5	4	14.8	66.6
Gynaecology	5	9.6	3	11.1	60
Orthopaedics	1	1.9	0	0	0
Radiology	0	0	0	0	0
Obstetrics	15	28.8	9	33.3	60
Paediatrics	1	1.9	1	3.7	100
Psychiatry	3	5.8	1	3.7	33.3
Other	11	21.2	7	25.9	63.6

Judicial and patient/defendant outcomes

Judicial Outcomes

**Figure 2:**
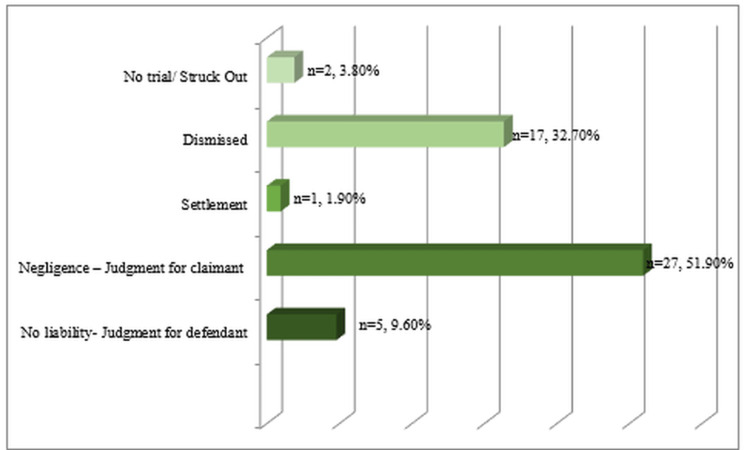
Lawsuit outcome for healthcare litigation cases (total n = 52)

The most common lawsuit outcome in the cases analysed was negligence/judgement in favour of the claimant (n = 27; 51.9%). Seventeen cases (32.7%) were dismissed, and in five cases (9.6%), there was no liability/judgement was awarded in favour of the defendant. Two cases (3.8%) did not reach trial, and one case (1.9%) arrived at a settlement (Figure 2). 

Patient/Defendant Outcomes

The most frequent physical patient outcome of medical negligence cases was death (n = 14; 26.9%), followed by temporary discomforts, such as repeated surgery and prolonged in-patient stays (n = 8; 15.4%). The most frequent monetary outcome was compensation for emotional harm (n = 14; 26.9%), followed by compensation for death/disability (n = 10; 19.2%). Only one case resulted in disciplinary action against the healthcare provider (n = 1; 1.9%) (Table 4).

**Table 4:** Patient/defendant outcome of healthcare litigation cases

Type of outcome			N	%
Monetary outcome		Compensation for death/disability	10	19.2
		Compensation for funeral expenses and the living expenses of dependents	2	3.8
		Compensation for emotional harm	14	26.9
		Damaged reputation/revocation of medical license/ loss of privilege to practise at one or more institutions/expulsion from medical association	0	0
		Disciplinary outcome	1	1.9
Patient outcomes	Physical	Death	14	26.9
		Loss of pregnancy/baby	3	5.8
		Temporary discomforts (e.g., repeated surgery, prolonged in-patient stay)	8	15.4
		Permanent injury (e.g., disability, loss of organ)	6	11.5
		Grave injury (e.g., brain injury)	5	9.6
		Other injury (e.g., chronic pain)	3	5.8
		Others	1	1.9
	Financial	Hospitalisation	0	0
		Loss of work	3	5.8
		Loss of future wages	4	7.7
		Others	0	0
	Psychological	Loss of quality of life	4	7.7
		Emotional burden (anxiety, depression, stress, insomnia, post-traumatic sress disorder)	5	9.6
		Trauma	1	1.9
		Other	0	0

Judicial Clinical Information

Judicial reviews revealed that most medical negligence cases resulted from clinical judgement errors, such as treatment overdosage (see Appendix A). An example is the Keda Lewis vs. Tobago Regional Health Authority case, where medicine was over-administered to an infant. In this case, medical negligence resulted from difficulty interpreting the cardiotocography (CTG), failure to perform foetal blood sampling and the delayed decision to carry out a caesarean section. Other medical negligence cases were due to failure to recognise and treat opioid overdose; assigning junior doctors without supervision; failure to recognise ineffective intubation; delay in seeking senior assistance; failing to report, record, recognise and rectify injuries; failing to listen to the patient; mistaken administration of the drug Depo-Provera (DP) (instead of Gynodian Depot - GD); false imprisonment where there was failure to comply with the terms of the Mental Health Act for detention in hospital as seen in the case of Adrian Hinds vs. The Northwest Regional Health Authority; improper sterilisation; dental issues; untimely attention to patients; insisting against patients' wishes and coercion of consent; lack of appreciation of patient concerns, as in the case of Rana Ramlal vs. Southwest Regional Health Authority, in which ‘dismissing the patient’s complaints of foetal movements as impossible, a view that, by the evidence of her own expert witnesses, was clearly wrong’; and improper assessment of chronic pelvic pain leading to infertility. Inappropriate drug dosage and usage accounted for 18.5% of cases convicted (n= 5/27). Other inadequate care standards reported in one case each (3.7%) included improper blood transfusion procedures, poor sterilisation of surgical instruments and poor listening and communication. Numerous protocols have been provided to clarify how gaps should be addressed, such as monitoring and dealing with injections (narcotics), listening to and communicating with patients, guidelines for blood transfusion, guidelines for the sterilisation of surgical instruments, and prevention of medication overdosage (see Appendix A).

## Discussion

Patients were predominantly female (n = 29; 55.8%). This was comparable to another study where females accounted for 57% of patients [17]. Most malpractice took place in public health institutions (n = 32; 61.5%), which was observed in a 10-year study conducted in Egypt, where claims were higher in public hospitals than in private hospitals [18]. Most claimants (n = 32; 61.5%) were patients, while the defendants were mainly healthcare institutions (n = 30/50 cases; 60%).

The healthcare institutions and medical personnel, particularly doctors, were mostly culpable (51.9% and 37%, respectively) because of delayed diagnoses/inadequate treatments (n = 23; 44.2%), misdiagnoses (n = 9; 17.3%), and prescribing the wrong dosages or medications (n = 7; 13.5%). This was comparable to a survey conducted by Schwarinzin [10], which revealed similar findings, where lawsuits were most commonly due to diagnosis and treatment errors. Though there may be a lack of professional adherence and medical incompetence [19], the public healthcare system lacks adequate resources [19] and enforcement by management to ensure guideline-based adequate care standards. Such operational issues have been reported by previous enquiry commissions, the last of which was the Gladys Gaffoor Commission of 2008 [20].

This study found that the largest category was comprised of obstetric cases. These findings were similar to those of Jean et al. [21], who found that litigation was highest for claims against obstetricians and gynaecologists. Out of the total number of cases convicted, the speciality that made up the majority of convicted cases was also that of obstetrics at 33.3%. Whilst obstetrics had the highest number of convicted cases, the conviction rate per speciality differs, with the highest conviction rate being in the paediatrics department (100%). Notably, it was only one case and should be considered with caution. Another study found the highest conviction rate (36.8%) in the emergency department [22]. In this study, the conviction rate arising from the emergency medicine department was still high, i.e., 66.6%. Furthermore, this study revealed that judgements were in favour of the claimant in at least 50% of cases. Additionally, at least one-third of cases (32.7%) were dismissed. This was far higher than other studies, which found that most claims were successfully defended [23-25].

The most frequent outcomes of monetary compensation arose from death/disability (n = 10; 19.2%) and emotional harm (n = 14; 26.9%). In Bishop et al. [26], compensation for death occurred in 30.4% of paid claims, while compensation for emotional harm was rare and accounted for only 1% to 2% of paid claims. This was in stark contrast to the present study, where emotional harm comprised the majority of cases of monetary compensation. Hence, emotional harm is considered when deciding liability, as it has been suggested that both physical and emotional injuries are valid types of harm [27]. Temporary discomforts (e.g., repeated surgery, prolonged inpatient stays) featured in a fair number of cases as well (n = 8; 15.4%). Appropriate standards of care reported by professional experts included proper history documentation, double-checking drug usage/dosage, calling for help and following procedures, such as the transfusion protocol, swab count, monitoring (perforated bowel), communication, safety protocol, and timely intervention. Many of the operating procedures and care standards were highlighted in reports by the WHO and the Gladys Gaffoor Commission [28,29]. The court cases led to practice guidelines being incorporated by judges in their decision-making process to increase adherence to care standards and improve the quality of care [30].

This study has certain limitations which should be considered in future studies. The sample size used in this study is small after the application of admission criteria. In addition, certain figures must be interpreted with caution. For instance, while the conviction rate in paediatrics was 100%, there was only one paediatric case out of the 52 cases used, and this one case resulted in a conviction. Ideally, a larger sample size is required to improve statistical reliability. Furthermore, court cases alone may not be representative of medical negligence. This is because an unknown number of cases never reach courts due to patients’ unawareness, resource constraints, out-of-court settlements, limited evidence, clients’ personalities, and judicial inefficiencies, such as long waiting periods. Additionally, this study was restricted to one location. Whilst Trinidad and Tobago share many similarities to other Caribbean Community and Common Market (CARICOM) nations with regard to healthcare and legal systems, future studies should consider other geographies to test whether these findings are generalisable. Other sources such as hospital records, media reports, social media forums and support groups can be included to understand sources of possible litigation and a probable finding of medical negligence in a court of law.

## Conclusions

Most medical negligence cases occurred in public institutions, particularly in obstetric departments, followed by emergency departments. Although health institutions accounted for most claims, doctors made up a significant proportion. Litigation mainly resulted from delayed diagnoses/inadequate treatments and breaching care standards. Greater efforts must be made by healthcare providers to identify the type, degree, and details of negligence through litigation analyses to drive quality improvement from an individual to an institutional level.

## Appendices

Appendix A

The medical negligence cases featured in this retrospective study are listed in Table 5.

**Table 5:** Titles of medical negligence cases TT HC: Trinidad & Tobago High Court, TT CA: Trinidad & Tobago Court of Appeal

Case no.	Case title
A1	Keda Lewis v Tobago Regional Health Authority. 2023, Claim No. CV2020-02315 (Trinidad & Tobago High Court (TT HC))
A2	Suzanne Dickson v Medcorp Ltd. 2022, Claim No. CV2015-01556 (TT HC)
A3	Stacey Campbell v Northwest Regional Health Authority. 2022, Claim No. CV2020-01194 (TT HC)
A4	Lorne Ramsoomair v South West Regional Health Authority; Seenath M; Ramballack D; Mohammed A. 2013, Claim No. CV2012-01864 (TT HC)
A5	Linda Rajkumarsingh v Gulf View Medical Centre Ltd. 2012, Claim No. CV2010-01958 (TT HC)
A6	Adrian Hinds v Northwest Regional Health Authority. 2017, Claim No. CV2017-00489 (TT HC)
A7	Cooper v Ramessar. 1974, No. 592 of 1974 (TT HC)
A8	Gosine v Dental Board of Trinidad and Tobago. 1995, CA MAG 190/1992 (Trinidad & Tobago Court of Appeal (TT CA))
A9	Southwest Regional Health Authority v Samdaye Harrilal. 2011, CV App. No. 60 of 2008; HCA No. 555 of 2003 (TT CA)
A10	Rana Ramlal v Southwest Regional Health Authority; Deonarine P. 2003, No. 1291 of 1998 (TT HC)
A11	Paula Mapp Joseph; Hugh Joseph v Minister of Health; NorthWest Regional Health Authority. 2007, Claim No. CV2006-03205 (TT HC)
A12	Angela Badree v NorthWest Regional Health Authority. 2017, Claim No. CV2017-00174 (TT HC)
